# Optimizing osteochondral decalcification and histological processing in ovine joint models

**DOI:** 10.29374/2527-2179.bjvm013925

**Published:** 2026-08-03

**Authors:** Natália Camargo Faraldo, Marcela dos Santos Ribeiro, Emanuel Vitor Pereira Apolonio, Lorena Cardozo Ferrari, Lucas Cirelli Portugal, Fernanda Barthelson Carvalho de Moura, Ana Liz Garcia Alves, Carlos Eduardo Fonseca-Alves

**Affiliations:** 1 Departamento de Cirurgia Veterinária e Reprodução Animal, Faculdade de Medicina Veterinária e Zootecnia, Universidade Estadual Paulista Júlio de Mesquita Filho (UNESP), Botucatu, SP, Brazil.; 2 Departamento de Cirurgia Veterinária, Faculdade de Medicina Veterinária e Zootecnia, Universidade de São Paulo (USP), São Paulo, SP, Brazil.

**Keywords:** decalcification, ovine joint, histology, orthopedic pathology, nitric acid, translational model, descalcificação, articulação ovina, histologia, patologia ortopédica, ácido nítrico, modelo translacional

## Abstract

Decalcification is a critical step in bone and cartilage histology that directly influences tissue preservation and staining quality. This study aimed to establish an efficient decalcification protocol for ovine stifle joint tissues while optimizing their morphological preservation and staining performance. Samples from the distal femoral condyles of four sheep were allocated to three experimental groups according to the decalcification protocol: 2% ethylenediaminetetraacetic acid (EDTA), 0.1% nitric acid, and a combination of both reagents. Samples were evaluated at different time points for mineral removal, preservation of cellular and tissue architecture, and staining quality using hematoxylin and eosin and Safranin O/Fast Green techniques. Histological preservation was assessed using the O’Driscoll scoring criteria. The 0.1% nitric acid protocol demonstrated the best balance between decalcification efficiency and tissue preservation, achieving satisfactory mineral removal within 48 h while maintaining cartilage and subchondral bone morphology. In contrast, EDTA requires longer decalcification periods (5–7 days), resulting in slower mineral removal and mild, progressive loss of cellular detail over time. The combined EDTA–nitric acid protocol promoted rapid decalcification but caused substantial tissue degradation, reducing histological quality. Histological evaluation confirmed that the preservation of hyaline cartilage architecture, chondrocyte morphology, extracellular matrix staining, and osteochondral organization was superior in the nitric acid group. These findings indicate that 0.1% nitric acid is a reliable and time-efficient protocol for decalcifying ovine osteochondral tissues, enabling adequate histological evaluation while preserving tissue integrity. This study provides a practical methodological reference for the histological processing of ovine joint tissues in experimental and translational research settings.

## Resumo

A descalcificação é uma etapa crítica na histologia óssea e cartilaginosa, influenciando diretamente a preservação do tecido e a qualidade da coloração. Este estudo teve como objetivo estabelecer um protocolo eficiente de descalcificação para tecidos da articulação do joelho de ovinos, otimizando a preservação morfológica e o desempenho da coloração. Amostras do côndilo femoral distal de quatro ovelhas foram divididas em três grupos experimentais de acordo com o protocolo de descalcificação: ácido etilenodiaminotetracético (EDTA) a 2%, ácido nítrico a 0,1% e uma combinação de ambos os reagentes. As amostras foram avaliadas em diferentes momentos quanto à remoção de minerais, preservação da arquitetura celular e tecidual e qualidade da coloração, utilizando as técnicas de hematoxilina e eosina (H&E) e Safranina O/Fast Green. A preservação histológica foi adicionalmente avaliada utilizando os critérios de pontuação de O’Driscoll. O protocolo com ácido nítrico a 0,1% demonstrou o melhor equilíbrio entre eficiência de descalcificação e preservação tecidual, alcanando remoção satisfatória de minerais em 48 horas, mantendo a morfologia da cartilagem e do osso subcondral. Em contraste, o EDTA exigiu períodos de descalcificação mais longos (5–7 dias), resultando em remoção mais lenta de minerais e uma perda leve e progressiva de detalhes celulares ao longo do tempo. O protocolo combinado de EDTA e ácido nítrico promoveu uma descalcificação rápida, mas causou degradação tecidual substancial e reduziu a qualidade histológica. A avaliação histológica confirmou que o grupo tratado com ácido nítrico proporcionou uma preservação superior da arquitetura da cartilagem hialina, da morfologia dos condrócitos, da coloração da matriz extracelular e da organização osteocondral. Esses achados indicam que o ácido nítrico a 0,1% é um protocolo confiável e eficiente em termos de tempo para a descalcificação de tecidos osteocondrais ovinos, permitindo uma avaliação histológica adequada ao mesmo tempo em que preserva an integridade do tecido. O estudo fornece uma referência metodológica prática para o processamento histológico de tecidos articulares ovinos em contextos de pesquisa experimental e translacional.

## Introduction

Articular cartilage injuries considerably affect quality of life by causing pain and impairing mobility (Kresakova et al., 2021; Le et al., 2020). Microlesions induced by biomechanical overload or degenerative diseases initiate an inflammatory process within the joint environment, with the release of cytokines, matrix metalloproteinases (MMPs), and hyaluronidases into synovial fluid. These mediators exacerbate inflammation and contribute to the progressive degradation of the cartilage matrix (Goldring & Otero, 2011; Kresakova et al., 2021; Le et al., 2020; Makris et al., 2015; Martel-Pelletier et al., 2016). Animal models have been widely used to study regenerative therapies for articular cartilage. Large animal models, including pigs, goats, horses, and sheep, have been used for this purpose (Music et al., 2018). Among these, sheep are particularly valuable because of their structural, biochemical, physiological, and immunological similarities to humans, as well as the feasibility of applying surgical protocols and postoperative care comparable to those used in clinical settings (Pilichi et al., 2014). In this context, sheep represent a robust translational model, as their articular cartilage thickness, zonal architecture, and biomechanical loading are similar to those of the human knee, enabling a more realistic assessment of the efficacy and integration of the proposed therapies (Music et al., 2018; Pilichi et al., 2014).

Articular cartilage consists of hyaline cartilage and contains chondrocytes responsible for synthesizing and maintaining the extracellular matrix, which is predominantly composed of type II collagen and proteoglycans, such as aggrecan (Eftekhari et al., 2020; Goldring & Goldring, 2007; Huey et al., 2012; Kresakova et al., 2021; Makris et al., 2015; Malemud, 2017; Newman, 1998). The regeneration of articular cartilage is limited by its lack of vascularization, which hinders the delivery of growth factors and stem cells to the injury site (Eftekhari et al., 2020; Goldring & Goldring, 2007; Hunziker, 2002; Kresakova et al., 2021). Avascular tissues rely on nutrient diffusion from the synovial fluid for cellular maintenance and metabolite exchange. Furthermore, because articular cartilage is aneural, degradation occurs asymptomatically until it reaches the subchondral bone, a richly innervated structure responsible for the onset of pain (Huey et al., 2012; Malemud, 2017).

Various decalcifying agents are used for the histological assessment of osteochondral lesion repair. These agents are employed to remove mineral content from bones, yet there is a paucity of discourse concerning their effects on tissue quality during histochemical staining (Broomfield et al., 2022; Liu et al., 2017). Liu et al. (2017) evaluated various decalcifying agents for their effectiveness in removing mineral content from rat femurs and examined their effects on tissue quality through hematoxylin and eosin (H&E) and immunohistochemical staining. Studies have shown that the choice of decalcifying solution significantly affects sectioning ease, morphological clarity, and antigenicity, highlighting the importance of selecting an appropriate decalcification method according to the analytical goal—structural evaluation or molecular detection—to ensure reliable and high-quality histological outcomes (Broomfield et al., 2022; Liu et al., 2017).

Although numerous methods have been published for bone decalcification via paraffin histology, detailed protocols specifically addressing the processing of ovine osteochondral tissue are lacking. Ovine bones present particular challenges, as specimens are often denser and larger than those obtained from other commonly used animal models (Broomfield et al., 2022). One study aimed to identify an optimal decalcification method for ovine humeral bone tissue that preserves cellular details, particularly in samples containing soft tissue attachments, such as tendons and spinal discs. Given the density and size of ovine bones, standard decalcification methods often result in tissue degradation (Broomfield et al., 2022). Thus, the present study aimed to propose an optimal decalcification technique for assessing ovine stifle osteochondral fragments that preserves tissue structure and facilitates histological examination. Therefore, this study aimed to evaluate and optimize decalcification protocols for ovine osteochondral tissues, focusing on improving histological processing and morphological preservation rather than investigating pathological alterations.

## Materials and methods

### Ethical approval

This study was approved by the Committee on Animal Research of São Paulo State University (0431/2023).

### Sample collection and morphological analysis

The samples used in this study were obtained from the Veterinary Surgery Service and sent to the Veterinary Pathology Laboratory for further analyses. We examined the stifle joints of four ovines (OV1-4) that underwent arthrotomy at São Paulo State University (UNESP, Brazil) in 2024 (study design). The samples were fixed in 10% formalin for 24–48 h. The femoral condyle samples showed no macroscopic alterations (König & Liebich, 2020; Sisson et al., 1976). The articular cartilage was smooth, shiny, and intact (Figure 1). The subchondral bone architecture was well preserved (Figure 1).

**Figure 1:**
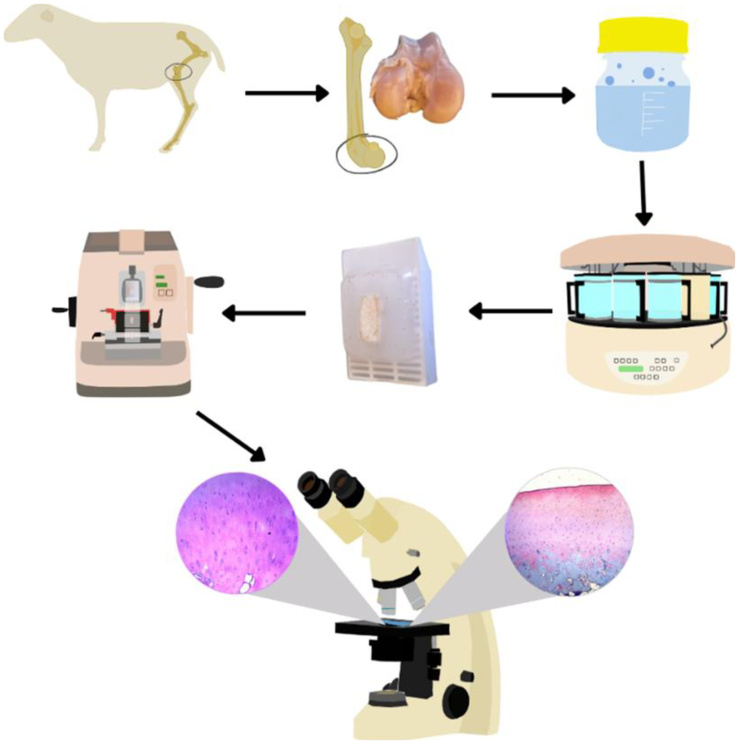
Schematic representation of the study design.

### Decalcification time-specific methods

The fragments of the four ovine specimens were randomly allocated into three experimental groups according to the decalcification protocol used. Group 1 was subjected to decalcification with 2% EDTA solution and evaluated daily for up to 7 days (168 h). Group 2 was subjected to decalcification with 0.1% nitric acid solution and evaluated at 12, 24, 48, 60, and 72 h. Group 3 was subjected to a combined protocol consisting of initial exposure to 2% EDTA followed by 0.1% nitric acid solution, with evaluations performed at the same time intervals (12, 24, 48, 60, and 72 hours).

To ensure standardization, all fragments were maintained under identical processing conditions with respect to solution volume, temperature, and fixation time. The decalcification endpoint was determined on the basis of mechanical testing using a needle and the histological processing quality. To mitigate this risk and preserve tissue integrity, needle probing in our study was performed only on the peripheral trimming zones (the outermost edges of the fragments) that were discarded during microtomy. The central region of interest designated for histological analysis remained untouched and free of any mechanical artifacts. Adequate decalcification was defined according to the following criteria: (i) the ability to obtain continuous 4 μm paraffin sections without tissue tearing or blade damage during microtomy; (ii) preservation of tissue architecture, including bone trabeculae and articular cartilage integrity; and (iii) satisfactory histochemical staining performance in H&E and safranin O/fast green preparations without excessive tissue fragmentation or loss of cellular detail.

After decalcification, the samples were transferred to 70% ethanol and routinely processed for paraffin embedding and microscopic evaluation.

### Histological assessment

For histological analysis, 4 μm sections were cut from the paraffin blocks using a semiautomatic microtome (Leica Biosystems, Wetzlar, Germany) and spread on histological slides (Erviegas, São Paulo, SP, Brazil). The slides were stained with H&E and safranin O/fast green for better structural characterization and possible detection of bone tissue lesions in the studied species. H&E staining was performed according to the laboratory protocol. Safranin O/Fast Green staining was performed according to the protocol established by the Rochester Medical Center (University of Rochester Medical Center, 2026). H&E staining was used to identify cellular components, whereas safranin O/fast green staining was used to assess the presence of a cartilaginous matrix (Figure 1).

The slides were examined using an optical microscope (Leica Biosystems, Wetzlar, Germany). The evaluation was conducted at magnifications of 100x, 200x, and 400x. Cartilage integrity was assessed using the criteria established by the O’Driscoll scoring system (O’Driscoll et al., 1988), which is typically applied in joint histopathology.

### Study design

The samples were subjected to different decalcification protocols commonly employed in routine histopathological laboratories and previously described in the literature for mineralized tissues. Ovine femoral condyle fragments were subcategorized into experimental groups according to the decalcifying solution used. The groups were compared in terms of decalcification time, tissue-processing feasibility, bone and cartilage morphology preservation, and the quality of histological staining using H&E and safranin O/fast green (Figure 1) (Broomfield et al., 2022; Charnwichai et al., 2023).

The evaluation focused on preserving microscopic structural integrity, including cellular morphology, trabecular organization, cartilage matrix staining, and the absence of processing artifacts that could compromise histological interpretation (Figure 1).

### Study limitations

This study has some limitations that should be considered when interpreting its results. First, the sample size was limited because only four ovine femoral condyle specimens were evaluated. In addition, this study focused exclusively on healthy ovine articular tissues and did not include samples affected by degenerative or inflammatory joint diseases, which may present different decalcification behaviors.

Another limitation is that the evaluated protocols were restricted to decalcifying agents routinely available in our laboratory, and other commercially available or advanced decalcification methods were not investigated. Furthermore, although the histological quality and staining preservation were systematically assessed, quantitative assessments of residual calcium content were not performed.

It is also important to recognize that decalcification protocols for mineralized tissues have already been widely described in the literature. Thus, the present study does not propose a novel decalcification technique but rather provides a comparative application of established protocols in an ovine femoral condyle model, emphasizing their practical implications for routine veterinary histopathology and cartilage evaluation.

## Results

The 2% EDTA protocol provided good preservation of tissue morphology throughout the experimental period. The histological architecture, including the osteochondral junction and cartilage matrix organization, remained well preserved during the first 5 days. However, decalcification progressed slowly, with high residual calcium intensity observed between days 1 and 4, moderate intensity on day 5, and low intensity on days 6 and 7. Although tissue integrity was largely maintained, prolonged exposure to EDTA was associated with a progressive loss of cellular detail, which became evident on day 6 and was more pronounced on day 7 (Table 1). At earlier time points, incomplete mineral removal impaired microtomy, preventing adequate sectioning and histological evaluation in some samples. These findings demonstrate that while EDTA effectively preserves tissue morphology, its slow decalcification rate may limit its practical applicability in routine diagnostic workflows that require rapid tissue processing.

**Table 1:** Macroscopic morphology preservation, calcium intensity, and loss of cellular detail of the articular cartilage and subchondral bones following different methods of decalcification (Broomfield et al., 2022; Charnwichai et al., 2023).

**Method of decalcification**	**Timing of decalcification (days)**	**Macroscopic morphology preservation**			**Calcium intensity**			**Loss cellular detail (score)**					
		**Poor**	**Fair**	**Good**	**Low**	**Moderate**	**High**	**0**	**1**	**2**	**3**	**4**	**5**
**2% EDTA**	1			X			X	N/E					
	2			X			X	N/E					
	3			X			X						
	4			X			X						
	5			X			X						
	6			X		X						X	
	7			X	X								X
**0.1% Nitric acid**	1			X			X	N/E					
	2			X		X		N/E					
	3			X		X		X					
**2% EDTA + 0.1% Nitric acid**	1			X			X	N/E					
	2		X			X						X	
	3	X			X								X

N/E, not evaluated (these sections were too hard and not suitable for sectioning and histological processing).

In contrast, the 0.1% nitric acid protocol achieved substantially faster decalcification. By 48–72 h, the samples exhibited moderate residual calcium intensity while maintaining satisfactory tissue integrity and histological quality. The osteochondral architecture, adequate staining quality, and minimal loss of cellular detail were preserved in the nitric acid group during the evaluation period. Histological sections revealed a smooth articular surface composed of hyaline cartilage overlying the preserved subchondral bone, with clear visualization of chondrocytes, collagen organization, and extracellular matrix staining (Figures 2 and 3). Safranin O/Fast Green staining also demonstrated the preservation of the proteoglycan-rich cartilage matrix in this group.

**Figure 2:**
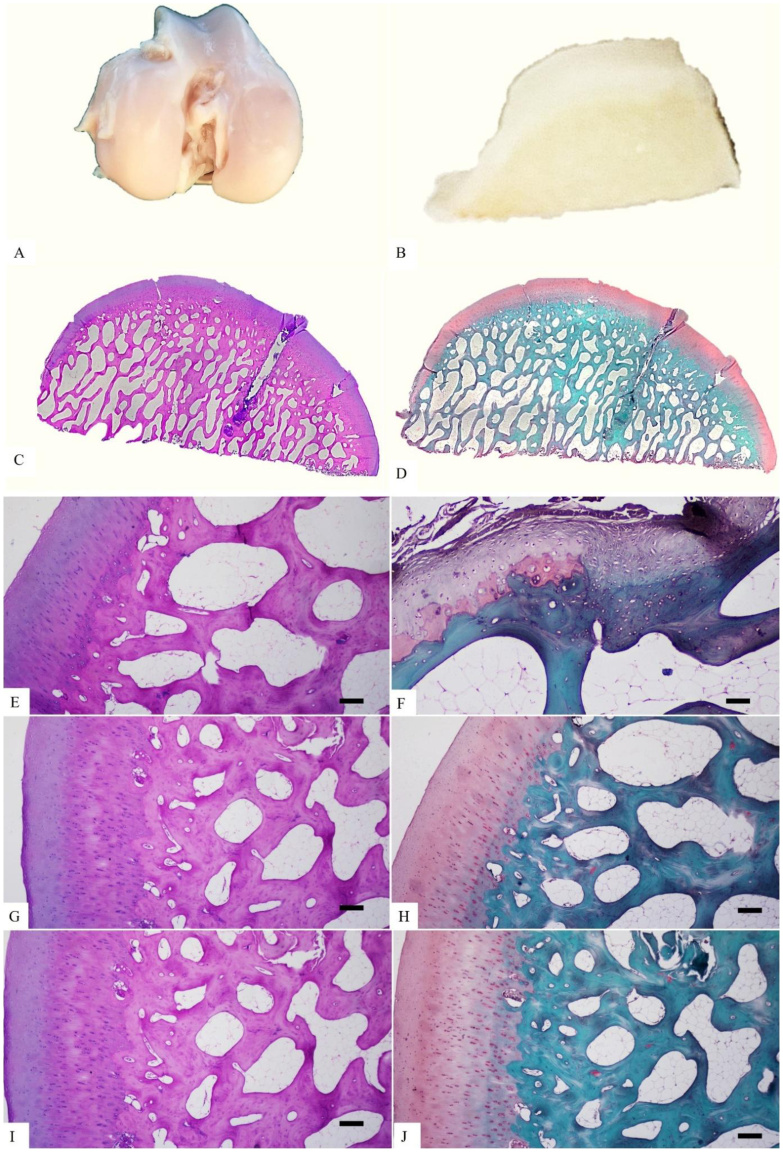
Gross and histological appearance of the normal distal femoral articular surface of an ovine stifle joint. A. Formalin-fixed distal femur fragment. B. Cross-section of the formalin-fixed specimen from the second decalcification group showing the preservation of the osteochondral junction morphology. C. Cross-section of hematoxylin and eosin-stained specimen from the second decalcification group. D. Cross-section of the safranin O/fast green-stained specimen from the first decalcification group. E, G, and I. Hematoxylin and eosin staining; scale bar = 100 µm, 4x. B, D and F, H and J. Safranin O/Fast Green Stain, Scale bar = 100 µm, 4X.

**Figure 3:**
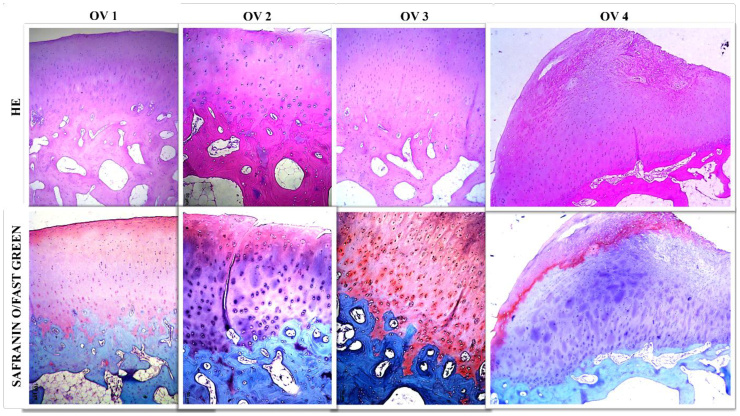
Representative images from the four ovines (OV1–OV4) showing the preservation of osteochondral architecture after decalcification. Upper row: Hematoxylin and eosin staining showing the articular cartilage surface, chondrocyte distribution, and subchondral bone organization. Lower row: Safranin O/Fast Green staining demonstrating the preservation of the proteoglycan-rich cartilage matrix and osteochondral junction. The nitric acid protocol at 48 h provided satisfactory preservation of tissue morphology and staining quality, allowing clear visualization of the cartilage and subchondral bone structures. Bar = 100 µm, 4x.

Among the tested methods, the nitric acid protocol provided the most favorable balance between decalcification efficiency and preservation of histological quality. These findings suggest that low concentrations of nitric acid may be suitable for routine processing of ovine osteochondral tissues when a rapid turnaround time is needed. Nevertheless, these findings should be interpreted cautiously, as only qualitative histological parameters were assessed without quantitative molecular or biochemical evaluation of tissue preservation.

The combined EDTA + nitric acid protocol exhibited the poorest overall performance. Although mineral removal occurred rapidly, tissue preservation deteriorated considerably after the first day. Histological evaluation revealed marked structural disruption, loss of cellular definition, and compromised staining quality, particularly after prolonged exposure. By the third day, severe tissue degradation prevented reliable histological interpretation in most sections (Table 1). These findings indicate that the sequential use of EDTA and nitric acid may have cumulative deleterious effects on osteochondral tissues, limiting their usefulness in studies requiring the preservation of cartilage morphology and matrix organization.

The comparative findings across the three protocols reinforce the importance of balancing the decalcification rate with tissue preservation. Faster protocols improve laboratory workflows but increase the risk of tissue damage. Thus, the optimal protocol should be selected according to the intended histopathological application and degree of structural preservation needed. The O’Driscoll scoring system was applied only to samples that fulfilled the minimum histological criteria for reliable evaluation, including adequate sectioning quality and preservation of the cartilage architecture.

Samples obtained at early time points in the EDTA and combined protocol groups were excluded from scoring because incomplete decalcification or severe tissue degradation impaired accurate assessment. In samples obtained at 48 h in the nitric acid group, the O’Driscoll scores were consistent with the qualitative histological observations, demonstrating the preservation of cartilage morphology, matrix staining, and structural organization (Table 2).

**Table 2:** O’Driscoll scoring system for the healthy ovine stifle joint specimen obtained within 48 h in the nitric acid group (O’Driscoll et al., 1988).

**Category**	**Score Range**	**Criteria**	**Score Given**
**Cell Morphology**	0–4	4 = Hyaline cartilage-like	4
		3 = Mixed hyaline and fibrocartilage	
		2 = Predominantly fibrocartilage	
		1 = Mostly noncartilaginous tissue	
		0 = Noncartilaginous tissue	
**Matrix Staining (Proteoglycan Content)**	0–3	3 = Normal	3
		2 = Slight reduction	
		1 = Marked reduction	
		0 = No staining	
**Surface Regularity**	0–3	3 = Smooth and intact	3
		2 = Slight fibrillation	
		1 = Moderate irregularities	
		0 = Severely irregular	
**Structural Integrity**	0–3	3 = Normal structure	3
		2 = Moderate disorganization	
		1 = Severe disorganization	
		0 = Complete disintegration	
**Thickness of Repair Tissue**	0–2	2 = Normal thickness	2
		1 = Slightly thinner or thicker	
		0 = Severely thinner or thicker	
**Integration with Adjacent Cartilage**	0–2	2 = Complete integration	2
		1 = Partial integration	
		0 = No integration	
**Freedom from Degenerative Changes in Adjacent Cartilage**	0–3	3 = Normal	2
		2 = Slight changes	
		1 = Moderate changes	
		0 = Severe changes	

Although the O’Driscoll system is traditionally used for cartilage repair assessment, in the present study, it served as an auxiliary histological parameter to support the evaluation of tissue preservation following decalcification. Therefore, O’Driscoll scores should not be interpreted as evidence of orthopedic or pathological alterations but rather as an indicator of histological quality after tissue processing.

## Discussion

Orthopedic pathology is a valuable diagnostic tool for identifying lesions in bones and associated tissues. Decalcification, followed by histological processing of samples, is a crucial step in enabling accurate diagnosis of tissue alterations via microscopy (Anderson & Rings, 2009; Maxie, 2016). The decalcification protocols are well established in medicine (Broomfield et al., 2022; Liu et al., 2017). However, additional studies are needed in veterinary medicine to further advance this diagnostic domain, thereby enabling veterinarians to improve their diagnostic techniques and assisting clinicians and surgeons in managing orthopedic patients (Broomfield et al., 2022; Tienen et al., 2003). Pathological conditions that may be identified in ovine stifle joints include septic arthritis, osteoarthritis, and trauma (Anderson & Rings, 2009). Decalcification is a crucial step in the preparation of bone tissue for histological analysis, particularly when structural preservation is required (Broomfield et al., 2022; Liu et al., 2017).

Various decalcifying agents have been studied. One study evaluated the use of an EDTA ultrasonic cleaner in conjunction with a microwave, demonstrating effective and rapid decalcification of the articular cartilage and subchondral bone (Charnwichai et al., 2023). Another study reported that among four decalcifying solutions tested, 3% nitric acid was the most effective decalcifying agent for H&E staining (Liu et al., 2017). Our findings are consistent with those of previous studies . We observed that the nitric acid protocol yielded the best results at 12, 24, 48, 60, and 72 h. Fragments measuring 0.5, 1, and 3 cm in diameter required 12, 24, and 48 h, respectively, to achieve complete decalcification. The complete decalcification allowed the insertion of a needle measuring 0.25 × 0.7 mm into the bone without difficulty, visible damage, or fracture of the osteochondral structures. The O’Driscoll scoring system, developed for assessing human orthopedic lesions, can be applied to systematically evaluate the stifle joints of domestic species, as demonstrated in this study (O’Driscoll et al., 1988). Although originally developed to assess cartilage repair and regeneration, the O’Driscoll scoring system was used in this study as an auxiliary histological tool to evaluate tissue preservation after chemical decalcification. Rather than measuring regenerative outcomes, the score served as an indicator of morphological integrity, allowing for an objective assessment of cellular morphology, matrix staining, and structural organization after treatment with nitric acid or EDTA. Because successful decalcification should preserve tissue quality for subsequent histopathological analyses, maintaining adequate O’Driscoll scores provides evidence that the protocols preserved essential histological features. Therefore, the O’Driscoll score was used to assess the fidelity of tissue preservation after mineral removal rather than to evaluate tissue repair.

An important methodological aspect of this study is that not all samples were suitable for histological scoring using the O’Driscoll system. The proper application of this score depends on adequate tissue preservation and section quality, which were not consistently achieved across all decalcification protocols. In particular, incomplete decalcification or excessive tissue degradation impairs the evaluation of structural parameters required by the scoring system. These findings highlight that the effectiveness of a decalcification protocol should also be assessed in terms of its ability to generate samples compatible with downstream historical analyses. Sheep represent a robust translational animal model for evaluating the knee owing to their anatomical, physiological, and immunological similarities to humans, including comparable cartilage thickness, zonal architecture, and biomechanical loading, which allows for a realistic assessment of therapies under clinical-like conditions (Ahern et al., 2009; Little & Hunter, 2013; Oliveira et al., 2018; Peng et al., 2024).

This study underscores the importance of standardizing decalcification techniques for ovine stifle joints. Among the protocols evaluated, decalcification with 0.1% nitric acid solution was the most effective one for histological staining and subsequent evaluation of the stifle joint in sheep. Within the limitations of this study, the nitric acid protocol provided the most favorable balance between decalcification efficiency and preservation of tissue morphology, which was associated with adequate removal of mineral content and minimal loss of cellular detail, both of which are important for histological examination. A systematic approach to joint examinations can help detect lesions and improve ovine and human health.

An important aspect to consider is the balance between decalcification efficiency and tissue preservation. Although nitric acid demonstrated favorable performance in terms of processing time, rapid decalcification may increase the risk of tissue degradation depending on the combined EDTA‒nitric acid protocol, in which rapid mineral removal is associated with loss of structural integrity.

This study has some limitations that should be considered in future studies. The analysis was based on samples obtained from four animals, which limited the generalizability of our findings. Additionally, part of the evaluation relied on qualitative and semiquantitative criteria, which may have introduced observer-dependent variability. The O’Driscoll scoring system was applied to a limited subset of samples and was not designed for comparative statistical analyses across all experimental groups.

## Conclusion

Overall, the results support the conclusion that the evaluated decalcification methods have distinct effects on the preservation and processing quality of ovine osteochondral tissue. This study demonstrated that 0.1% nitric acid is an effective agent for decalcifying ovine osteochondral tissues, providing a favorable balance between processing time and preservation of tissue morphology. The O'Driscoll score was applied, with the maximum score assumed as the references for intact, healthy cartilage, thereby allowing quantification of structural loss or preservation. However, these findings should be interpreted within the limitations of the study design, particularly the small sample size and the absence of quantitative analytical methods for evaluating calcium removal and matrix preservation. Nevertheless, the findings provide practical methodological guidance for the histological preparation of ovine osteochondral specimens in veterinary research and diagnostic settings.
